# The framing of choice nudges prolonged processing in the evaluation of food images

**DOI:** 10.3389/fpsyg.2023.1039251

**Published:** 2023-06-09

**Authors:** Ji Xu, Yimeng Jin, Johan Lauwereyns

**Affiliations:** ^1^Graduate School of Systems Life Sciences, Kyushu University, Fukuoka, Japan; ^2^School of Interdisciplinary Science and Innovation, Kyushu University, Fukuoka, Japan; ^3^Faculty of Arts and Science, Kyushu University, Fukuoka, Japan

**Keywords:** choice framing, response time, prolonged processing, preference-based decision-making, naturalistic food images

## Abstract

Previous research suggests that the type of choice framing for evaluation tasks can influence the relationship between response time and preference-based decision-making. Two separable factors may modulate the preference-based decision-making: The set of choice options (with or without an option to defer) and the constraint of choice (with high or low maximum for inclusion). To clarify how these factors influence the process of preference-based decision-making, we designed a virtual-shopping paradigm with a series of food images presented consecutively, while varying the set of choice options and the constraint of choice. For the set of choice options, subjects were asked to choose for each food image in either a two-options condition (i.e., “take it” or “leave it”), or a three-options condition (i.e., “take it,” “wait,” or “leave it”). For the constraint of choice, subjects were instructed to select a maximum of either five items out of 80 (i.e., highly constrained) or 15 items out of 80 (i.e., less constrained). As in previous findings, the response times were consistently longer for “take it” than for “leave it” options. Importantly, this difference was exacerbated under high constraint, when subjects could select only five items, suggesting a role for opportunity-cost consideration in the decision process. Furthermore, as compared to two-options tasks, subjects consistently spent more time overall in the three-options tasks (with the option to defer), displaying lower acceptance rates, and particularly long response times for the “wait” option. This finding suggests that choice framing with a defer option nudges prolonged processing.

## Introduction

Arguably the most basic—and yet also most profound—statement we can make about the process of decision-making is that it takes time. Models of decision-making in psychology and neuroscience have focused on the dimension of time to characterize core phenomena such as the speed-accuracy tradeoff and the adaptive control of decision-making (e.g., Luce, 1963; Green and Swets, 1966/1988; Carpenter, 1981; Smith and Ratcliff, 2004; Bogacz et al., 2006; Gluth et al., 2012; Heitz, 2014; for a comprehensive review, see Vargas and Lauwereyns, 2021). Though most of this research focused on simple perceptual decision-making, the relevance of response time analysis extends to value-based decision-making and experimental economics (Spiliopoulos and Ortmann, 2018; Gluth et al., 2020). Prolonged response times may be indicative of deliberative decision-making (Van der Meer et al., 2012; Redish, 2016; Abram et al., 2019), with continued, effortful information processing, particularly when faced with difficult decisions. The information processing may include a range of cognitive operations such as perceptual feature extraction, outcome prediction, and opportunity cost consideration (Abram et al., 2019; Spiller, 2019; Couto et al., 2020; Vargas and Lauwereyns, 2021).

In the domain of value-based decision-making, the temporal analysis of behavior can be used to predict choice. The well-established gaze bias effect shows that subjects look longer at items they will choose than at items they will reject (Shimojo et al., 2003; Krajbich et al., 2010; Zommara et al., 2018; Thomas et al., 2019). However, the gaze bias effect is a phenomenon limited to forced-choice situations with two or more simultaneously presented options. This leaves open the possibility that prolonged viewing does not necessarily increase the likelihood of a positive evaluation in sequential preference-based decision tasks in which the decision-making does not involve spatial selection. The time invested for decision-making may reflect deliberative time allocation (Bhui, 2019) or uncertainty in the assessment of value, when it gradually becomes more difficult during sequential decision-making to stop or continue (Meder et al., 2016). Here, prolonged processing may be associated with information integration rather than the accumulation of positive value. To examine this possibility in our laboratory, Wolf et al. (2018, 2019) designed a set of two studies on sequential preference-based decision-making regarding naturalistic food images, without spatial selection.

In the two studies, Wolf et al. (2018, 2019) examined the role of task framing in the relationship between viewing and preference-based decision-making. In all trials, the stimuli were presented one-by-one at the same location at the center of the screen. For non-selective tasks, the subjects gave ratings without any limit on the number of positive ratings. In such tasks, the viewing and response times tended to be shorter for preferred items. For two-choice selective tasks, subjects had to make successive “take it” or “leave it” decisions to either include or exclude the presented item in the virtual shopping basket. In such tasks, the viewing and response times were longer for “take it” decisions. However, when the selective tasks were changed to a three-choice decision by adding an option to defer judgment (“wait”), the viewing and response times were the longest for the defer option.

Based on these findings, Wolf et al. (2018, 2019) concluded that the longer response times reflected an increased effort in information integration, not an inherent link between viewing and the accumulation of positive value. Here, we present a follow-up study to examine the conditions under which subjects may invest more time for information processing. We had two principal objectives. First, we aimed to replicate the finding that adding a defer option produces prolonged processing during decision-making. In the previous study, the two-choice tasks (“take it” or “leave it”) versus three-choice tasks with a defer option (“take it,” “wait,” or “leave it”) were tested between subjects. In the present study, the tasks were tested within subjects, examining whether the framing of choice with a defer option can effectively nudge the same subject toward prolonged processing. We predicted that, in the two-choice tasks, “take it” responses would be associated with longer response times than “leave it” responses. In the three-choice tasks, the “wait” responses would be associated with the longest response times.

Second, we hypothesized that the constraint of choice can lead to opportunity cost consideration. Subjects would consider their options more carefully when there is more selection pressure for the virtual shopping basket. Specifically, we compared a highly constrained choice task (to select up to five items from 80 sequentially presented options) against a less constrained choice task (to select up to 15 items). We predicted that the “take it” responses would be associated with longer response times than the “leave it” responses, particularly in highly constrained choice tasks.

## Methods

### Participants

A total of 64 subjects participated in this experiment. All were Kyushu University students (37 males, 27 females) with a mean age of 22.14 years old, and a standard deviation of 4.15. All subjects were naïve to the purpose of the experiment and had normal or corrected to normal vision. The study was conducted in accordance with the ethical principles of Kyushu University and approved by the Human Ethics Committee of the Faculty of Arts and Science. Each subject received either course credit or monetary compensation of 1,000 yen for their participation. Written informed consent was obtained from each subject before the experiment.

### Apparatus

All visual stimuli were presented on a 23.8-inch full high definition flat-panel-monitor, with a display resolution of 1920 × 1,080 pixels. To reduce head movement, a chin-rest with a forehead-support was used. The monitor screen was set approximately 65 cm from the chin-rest. A wireless keyboard was used for recording the manual responses. All events and recordings were controlled through code written in Psychopy (version 1.84.2). Subjects’ eye movements were recorded with an eye tracker, but not analyzed for this article.

### Stimuli

All visual stimuli were presented as inset images on a white background in the middle of the otherwise black screen. The stimuli were a set of 320 naturalistic food images and the size of each image was fixed at 600 × 450 pixels (96 dpi, standard Red Green Blue (sRGB) color format). The set of images was drawn from a food-pictures database (Blechert et al., 2014), and was identical to what we used in our previous studies (Wolf et al., 2018, 2019). The set of images was divided into four subsets of 80 pictures and applied to the four experimental conditions. The subsets were carefully balanced based on the values in the database of Blechert et al. (2014), obtained from four surveys with a total of 1,988 respondents. The values included objective characteristics reflecting information on macronutrients (e.g., protein per 100 g; fat per 100 g) and image characteristics (e.g., color, complexity), as well as subjective characteristics reflecting judgments of palatability and craving as a function of diet and gender. Statistical tests confirmed that there were no significant differences in any of the characteristics among the four subsets.

### Design and procedure

To clarify how the constraint of choice and the set of choice options influence the evaluative process, the experiment was designed by applying a virtual-shopping paradigm with a within-subjects design using different types of response framing. For the constraint of choice, there were two types of tasks with a different maximum of selections: a highly constrained task with a maximum of five selections (denoted as Max-5) or a less constrained task with a maximum of 15 selections (denoted as Max-15). For the set of choice options, there were two types of tasks: either a forced choice between “take it” or “leave it” (denoted as the Binary task) or a choice with three options, “take it,” “leave it,” or a third option to defer judgment, “wait” (this task with three options is denoted as the Defer task). All subjects participated in each of the four tasks, and in all tasks, the subjects were asked to pick food images for a virtual “basket” out of 80 images, which were presented consecutively, one by one. The option of “take it” means adding the food item into the virtual basket; conversely, “leave it” means the rejection of the item. The “wait” option implies the deferment of deciding for the presented item. To be more specific, the food items that were selected to the “wait” option would be excluded from the virtual basket once the subject reached the maximum of selections (i.e., five or 15).

Each trial started with a fixation for 1 s at the central fixation cross. After the fixation, a food image was presented at the center of the screen. The subjects were asked to look at the image and press the spacebar when they were ready to give their decision. On the choice screen, subjects were required to answer the question “Would you like to add this food image to your basket” by using the arrow keys. In the Binary task, the left and right arrow keys were available for “take it” or “leave it” respectively. In the Defer task, an up-arrow key representing the “wait” option was available in addition to the left and right arrow keys. The length of time to view the food image and give the decision was determined freely by the subject (i.e., self-paced). After each evaluation, a 2 s visual feedback was presented with the given choice and an update of the number of images selected and viewed. The inter-trial-interval was set for 1 s between the trials. Each task ended when the subject reached the maximum of selections (i.e., five or 15). The subjects were informed that in case they did not reach the maximum number of selections after viewing all 80 images, the items in the “wait” option would be manually presented again for selection. These second-round selections were not included in the analyses as they involved repeated exposure. The order of tasks was counterbalanced across subjects in four arrangements, ensuring that the Binary and Defer tasks were grouped together and the Max-5 and Max-15 tasks were alternated (see Table 1). The entire experimental session lasted approximately 1 hour.

**Table 1:** Scheme of counterbalancing with a within-subjects design.

Group	Block 1		Block 2		Block 3		Block 4	
	Decision	Max	Decision	Max	Decision	Max	Decision	Max
*Group 1* *N* = 16	Binary	5	Binary	15	Defer	5	Defer	15
*Group 2* *N* = 16	Defer	5	Defer	15	Binary	5	Binary	15
*Group 3* *N* = 16	Binary	15	Binary	5	Defer	15	Defer	5
*Group 4* *N* = 16	Defer	15	Defer	5	Binary	15	Binary	5

## Results

All analyses were based on the average measures per subject in each task. For each of the 64 subjects we recorded the decisions made: “Take it” or “leave it” in the Binary task; “take it,” “wait,” or “leave it,” in the Defer task. For each decision we recorded the response time. We also recorded the number of items viewed by the subjects in each task. All 64 subjects completed the four tasks. However, for the Defer tasks, a total of eight subjects never chose the “wait” option; one of these subjects also never chose the “leave it” option in the Max-5 task. This resulted in missing data for the analysis of response times as a function of choice. Consequently, we performed the analysis of response times in the Defer task based on 56 subjects, excluding the eight subjects with missing data for the “wait” option. In the analysis of the acceptance rates and in the correlation analysis for the Defer Max-5 task, we excluded the subject who never chose the “leave it” option. The other correlation analyses were based on the data from all 64 subjects.

For brevity, analyses of gaze duration and of between-group comparisons as a function of counterbalancing are not included here as they do not alter the present findings and interpretation.

### Response time

**Figure 1:**
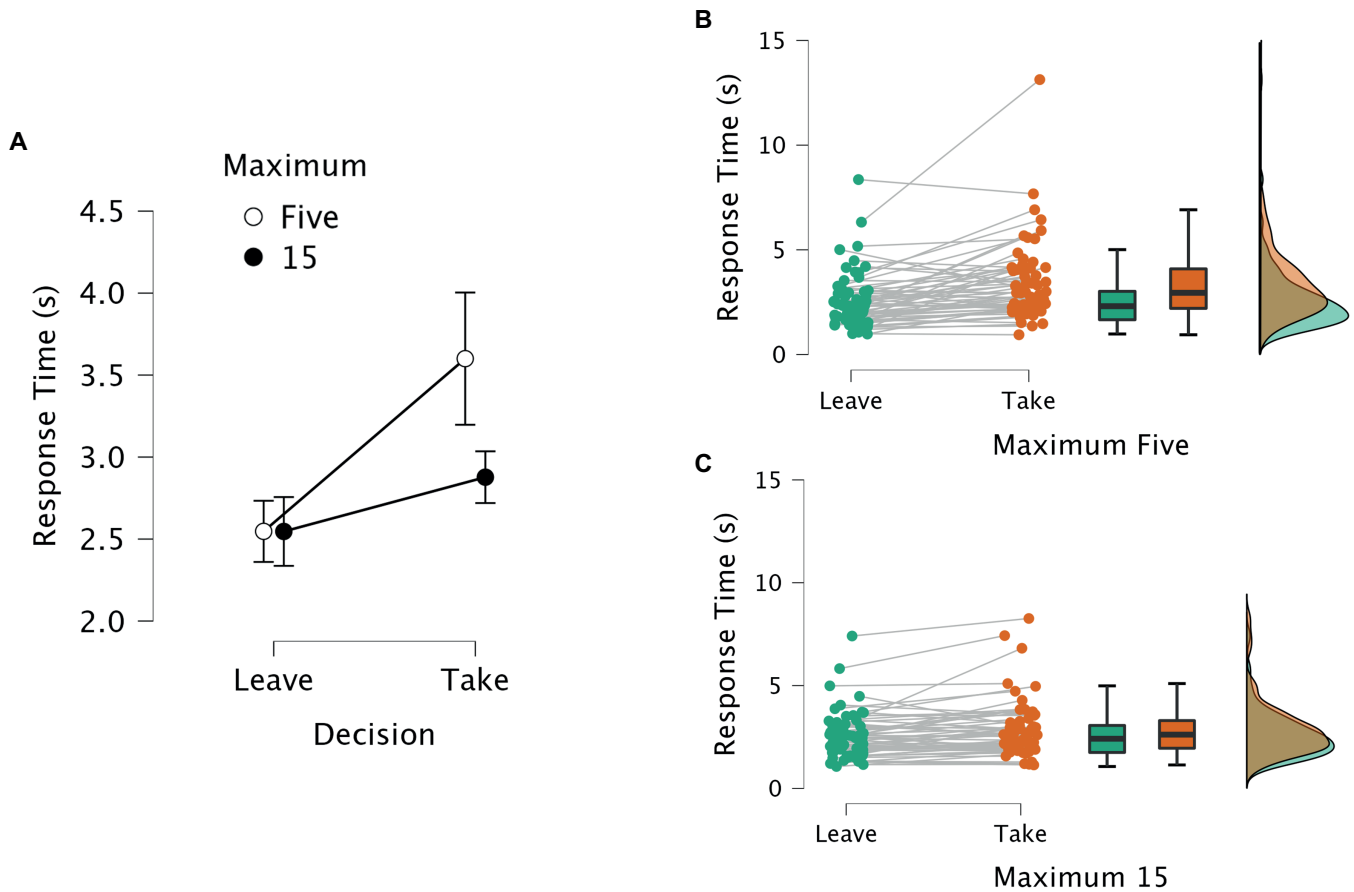
Response times in the Binary task. Panel **(A)** shows the average response times and 95% confidence intervals aggregated across subjects. Leave and Take reflect the options of “leave it” and “take it”; for each option, the unfilled data points stand for the response times in the highly constrained task (i.e., select a maximum of five items out of 80); the filled data points stand for the response times in the less constrained task (i.e., select a maximum of 15 items out of 80). Panels **(B)** and **(C)** provide raincloud plots with averages per subject, boxplots, and distribution density plots, with the Leave data in green and the Take data in orange. Panel B shows the data for the Max-5 task; Panel C shows the data for the Max-15 task.

**Figure 2:**
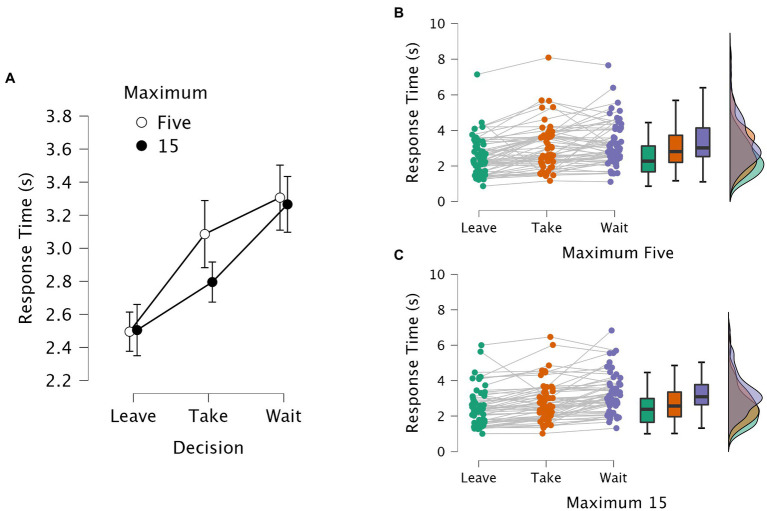
Response times in the Defer task. Panel **(A)** shows the average response times and 95% confidence intervals aggregated across subjects. Leave, Take, and Wait reflect the three choice options; for each option, the unfilled data points stand for the response times in the highly constrained task (i.e., Max-5); the filled data points stand for the response times in the less constrained task (i.e., Max-15). Panels **(B)** and **(C)** provide raincloud plots with averages per subject, boxplots, and distribution density plots, with the Leave data in green, the Take data in orange, and the Wait data in purple. Panel B shows the data for the Max-5 task; Panel C shows the data for the Max-15 task.

The response time was calculated from the onset of the food image until the moment the subject pressed an arrow key to indicate the decision. Figure 1 shows the average response times in the Binary task; Figure 2 shows the average response times in the Defer task.

For the Binary task, a 2 × 2 repeated measures ANOVA on response times was performed to analyze the within-subjects effect of the choice (i.e., “take it” versus “leave it”) and the constraint (i.e., Max-5 versus Max-15). There were significant main effects for choice (*F* (1, 63) = 22.641, *p* < 0.001, η^2^_p_ = 0.264) and for constraint (*F* (1, 63) = 7.477, *p* = 0.008, η^2^_p_ = 0.106). The result also showed a statistically significant interaction between the two factors (*F* (1, 63) = 11.506, *p* = 0.001, η^2^_p_ = 0.154). Post-hoc pairwise comparisons using the Bonferroni correction indicated that, in the highly constrained task (i.e., Max-5), the response times for the “take it” option were significantly longer than for the “leave it” option at *p* < 0.001, whereas in the less constrained task (i.e., Max-15) there was no significant difference between the two options. In addition, the difference between Max-5 and Max-15 for “take it” option was also significant (*p* < 0.001), which suggests that the subjects spent more time making the decision of including the food in the highly constrained task. There was no difference between Max-5 and Max-15 for the “leave it” option.

For the Defer task, a 3 × 2 repeated measures ANOVA on response times was performed to analyze the within-subjects effect of the choice (i.e., “take it,” “leave it,” or “wait”) and the constraint (i.e., Max-5 versus Max-15). The ANOVA result produced a significant main effect of choice (*F* (2, 110) = 37.990, *p* < 0.001, η^2^_p_ = 0.409), whereas the effect of constraint was not significant (*F* (1, 55) = 1.684, *p* = 0.200, η^2^_p_ = 0.030). Post-hoc pairwise comparisons using the Bonferroni correction indicated that the response times for all three choice options (“take it,” “wait,” or “leave it”) were significantly different from each other (*p*s < 0.001). When examining the interaction between the two factors, Mauchly’s test indicated that the assumption of sphericity had been violated (*χ*^2^ (2) = 9.119, *p* = 0.010), and so we applied the Greenhouse–Geisser correction. The analysis showed a significant interaction between choice and constraint (*F* (1.731, 95.207) = 3.718, *p* = 0.034, η^2^_p_ = 0.063). Post-hoc pairwise comparisons using the Bonferroni correction showed that, compared to the response times for the “leave it” option, the times for the “wait” and “take it” options were both significantly longer (*p*s < 0.001) in the highly constrained task (i.e., Max-5), whereas there was no significant difference between the two options of “wait” and “take it” (*p* = 0.632). In the less constrained task (i.e., Max-15), however, the response times for the “wait” option were significantly longer than those for both the “leave it” and “take it” options (*p*s < 0.001), while there was no significant difference between the two options of “leave it” and “take it” (*p* = 0.115).

### Acceptance rate

Next, we analyzed the acceptance rates in the four tasks. The acceptance rate was calculated as the number of items selected divided by the number of items viewed. The number of items viewed reflected the total number of images that the subject viewed before reaching the maximum of selections. In case the subject did not reach the maximum of selections after viewing all 80 images, the acceptance rate was based on the number of items selected at that time, divided by 80.

**Figure 3:**
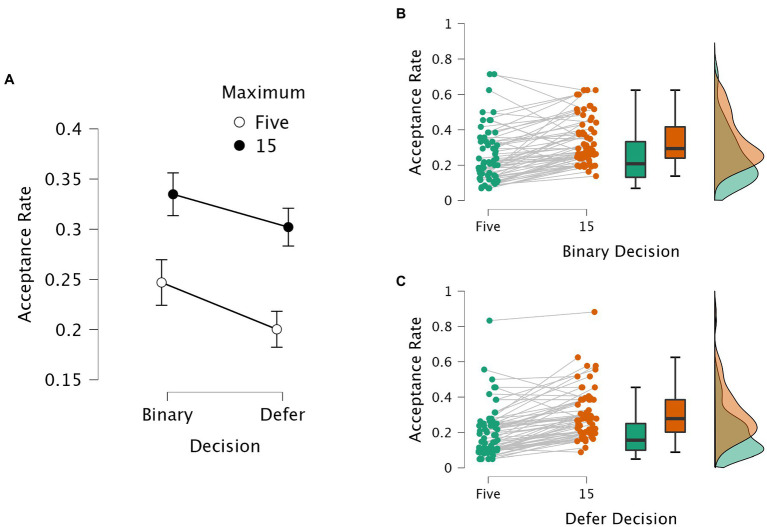
Acceptance rates in each task. Acceptance rate reflects the number of items selected divided by the number of items viewed. Panel **(A)** shows the average acceptance rates and 95% confidence intervals aggregated across subjects. Binary and Defer reflect the types of decision, without or with a defer option; the unfilled data points stand for the acceptance rates in the highly constrained task (i.e., Max-5); the filled data points stand for the response times in the less constrained task (i.e., Max-15). Panels **(B)** and **(C)** provide raincloud plots with averages per subject, boxplots, and distribution density plots, with the Max-5 data in green and the Max-15 data in orange. Panel B shows the data for the Binary task; Panel C shows the data for the Defer task.

Figure 3 presents the average acceptance rates in each task. A 2 × 2 repeated measures ANOVA was performed with the within-subjects factors decision task (i.e., Binary versus Defer) and constraint (i.e., Max-5 versus Max-15). The analysis showed that there were significant main effects for both the decision task (*F* (1, 62) = 14.888, *p* < 0.001, η^2^_p_ = 0.194) and the constraint (*F* (1, 62) = 69.219, *p* < 0.001, η^2^_p_ = 0.528), while there was no interaction between the two factors (*F* (1, 62) < 1). Compared to the Binary task, subjects produced lower acceptance rates in the Defer task; additionally, the acceptance rates were lower in the more constrained task (Max-5) as compared to the less constrained task (Max-15).

### Correlation analysis

To investigate the decision processes further, we conducted a correlation analysis to characterize individual tendencies among subjects. Particularly, subjects may differ with respect to the amount of time they take toward deciding to accept rather than to reject an item. Random or automatic decision-makers should show no difference in the response time to accept versus reject an item. However, longer response times for acceptance rather than rejection would reflect prolonged processing toward preference-based decision-making. For this purpose, we computed a differential response time, for each subject for each task, as (Take – Leave) / (Take + Leave), that is, the average response time for “take it” minus the average response time for “leave it,” normalized by the sum of the two average response times. Careful decision-makers would spend relatively more time before accepting an item. We hypothesized that such careful decision-makers would tend to display lower acceptance rates. If so, there should be a negative correlation between the differential response time and the acceptance rate.

**Figure 4:**
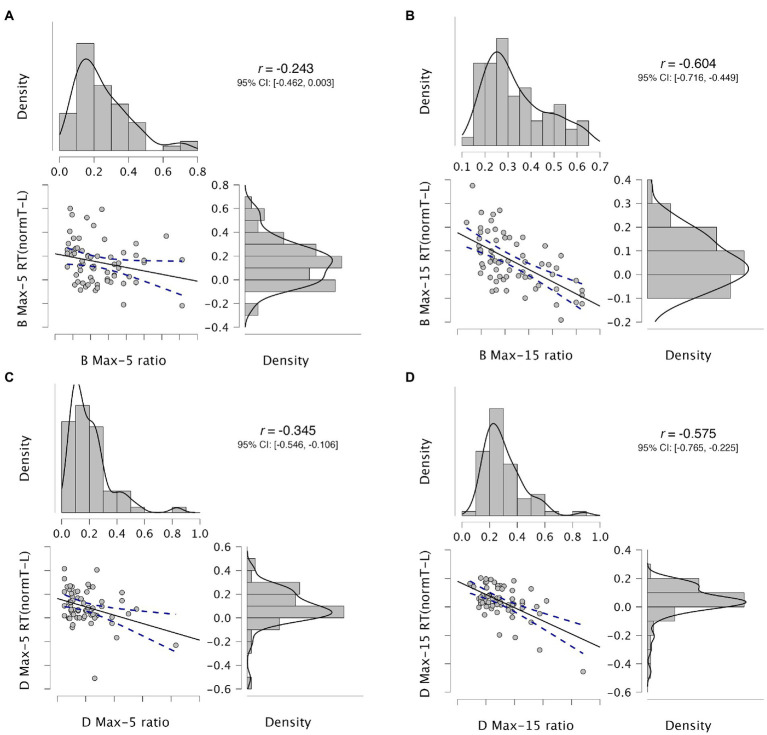
Correlations between acceptance rates and differential response times in each task. Acceptance rate reflects, for each subject for each task, the number of selected items divided by the number of viewed items. Differential response time is computed, for each subject for each task, as (Take – Leave) / (Take + Leave), that is, the average response time for “take it” minus the average response time for “leave it,” normalized by the sum of the two average response times. For each correlation, the horizontal dimension reflects the acceptance rate; the vertical dimension reflects the differential response time. Each correlation is represented with the scatterplot, the regression line, the 95% confidence region around the regression line, the density plots, the r value, and the 95% confidence interval of the r value. Panel **(A)** represents the Binary task with Max-5; Panel **(B)**, the Binary task with Max-15; Panel **(C)**, the Defer task with Max-5; and Panel **(D)**, the Defer task with Max-15.

For each task, we calculated a Pearson correlation coefficient to assess the relationship between the differential response time and the acceptance rate. Figure 4 shows the correlations for each of the four tasks, with each data point representing one subject in the scatterplots. The analysis showed that there was a strong negative correlation between the two variables in the Binary task with less constraint (Max-15, panel B), *r* = −0.604, *n* = 64, *p* < 0.001; whereas with high constraint (Max-5, panel A), the correlation was not significant (*r* = −0.243, *n* = 64, *p* = 0.053). In the Defer tasks, there were significant negative correlations for low constraint (Max-15, Panel D; *r* = −0.575, *n* = 64, *p* < 0.001) as well as for high constraint (Max-5, Panel C; *r* = −0.345, *n* = 63, *p* = 0.006). The results imply that subjects show individual differences with respect to the amount of time they invest toward preference-based decision-making. Those who show longer differential response times tend to be more careful decision-makers with lower acceptance rates in their virtual shopping tasks. Notably, the strength of this association varies as a function of the type of task, suggesting that the response framing can influence the length of time people take for making their choices.

## Discussion

In the present study, we systematically manipulated the choice framing with four versions of a virtual-shopping task in which subjects were asked to select their preferred items from a set of 80 naturalistic food images. The virtual shopping paradigm required sequential foraging with individually presented items. We manipulated the set of choice options, with either a Binary task in which subjects had to accept or reject each item in turn, or a Defer task in which subjects had a third option to defer judgment. We also systematically varied the constraint of the task, with either a strongly exclusive maximum of five items to be selected, or a less exclusive maximum of 15 items. Overall, we obtained clear evidence that the choice framing influenced the subjects’ processes of preference-based decision-making, both in the time spent processing the individual food images and in the number of images considered during foraging. The data corroborate and expand previous findings from our lab that the choice framing can influence the subject’s investment of time during preference-based decision-making (Wolf et al., 2018, 2019).

With respect to the set of choice options, we found that the inclusion of a defer option induced subjects to spend more time on the task overall, sampling more images, and taking the longest time processing images that ended up in the “wait” category. This finding confirms that viewing does not lead to the accumulation of positive value during serial preference-based decision-making, when items are presented one-by-one. The prolonged processing observed for the “wait” category implies a different function – not the accumulation of positive value, but information integration, where continued sampling does not necessarily imply an increased likelihood of preference.

Such an interpretation may well be compatible with Sequential Sampling Models (SSMs; e.g., Krajbich et al., 2010). Indeed, previous research has explicitly investigated the effect of the possibility to defer a choice using SSMs (Gluth et al., 2013; Hawkins and Heathcote, 2021). For example, the model in Gluth et al. (2013) assumes that on top of the accumulation toward the decision boundaries (in the current case, “take it” or “leave it”) a third accumulator joins the race to the thresholds and can terminate the accumulation process temporarily, if there is insufficient evidence for choosing one of the options. Such a model would be able to account for the particularly long “defer” decisions in the current task (the decision-maker only defers if there is weak evidence for “take it” or “leave it”). As a corollary, the explicit inclusion of a defer option may encourage people to gather more information before deciding.

With respect to the level of constraint of the task, we found that the more exclusive maximum of five items induced subjects to take more time before accepting an item. The response times were longer for the “take it” option than for the “leave it” option with a maximum of five items, though this extra processing was less pronounced with a maximum of 15 items. The added processing time in the highly constrained situation happened regardless of whether there was a defer option; the same result was found in the Binary and in the Defer task. One possibility is that the added processing time reflected opportunity cost consideration given the differences in risk faced by the subjects. Subjects did not know the full menu of choices before being required to take or leave a given option, implying the choice is made in the face of risk. Taking an option means risking a wrong choice, in case a preferred alternative would be shown later. This risk is higher in the Max-5 than in the Max-15 task. By this interpretation, the prolonged processing before deciding to “take it” reflected an extra check to ensure their positive evaluation of an item was strong enough to merit the exclusive spot in the virtual shopping basket. In terms of SSMs, this interpretation suggests that the prolonged processing reflects differences in the evidence accumulation.

An alternative interpretation would be that the shorter “leave it” than “take it” response times reflect a starting point bias toward the “leave it” threshold. This makes sense as there are more options to be shown than to be included in the virtual-shopping basket. For the same reason, the starting point bias should be even more pronounced when fewer options can be selected, explaining the particularly long response times for “take it” in the Binary task with maximum five items. The only difficulty for this interpretation is that the “leave it” response times were equally fast in the Binary Max-5 and Max-15 tasks (*cf.* Figure 1, Panel A), suggesting that the level of constraint caused a change not in the starting point but in the rate of evidence accumulation – unless, the “leave it” responses were affected by a physical limit, already as fast as they could be in the Binary Max-15 task. To gain a full understanding of the underlying decision processing, future work should apply the SSM framework to provide a parsimonious, mechanistic, and elegant account.

Importantly, in the present study, the effects of choice framing occurred even in a complete within-subjects design, by which each subject experienced each version of the virtual-shopping task within the space of an hour. Thus, the information processing for preference-based decision-making appears to be highly susceptible to manipulation, or nudging, with immediate changes as a function of the task context in the amount of time taken toward accepting rather than rejecting an item. Here, an emerging result from the RT distributions is that the “take it” responses tended to be bimodal in the Defer Max-5 task (see Figure 2B; orange), but not in the other tasks. One interpretation would be that this shape arises from individual differences (slow versus fast decision-makers) that emerge only under high constraint, with a defer option.

Our correlation analyses (see Figure 4) further underscore that the differential response times, while prone to significant interindividual variation, adapt to the type of task. A relevant avenue for future research, then, is to examine how subjects may be characterized on a scale of prolonged processing, with converging measures of the extent of image sampling and the extent of opportunity cost consideration. This should preferably be done with proper motivated-choice studies, to assess actual preferences rather than stated preferences. Such measures of prolonged processing might covary with individual susceptibility to (or resilience against) framing and nudging – a potential issue with important ethical implications, in addition to our core understanding of human decision-making.

## Data availability statement

The original contributions presented in the study are included in the article/Supplementary material, further inquiries can be directed to the corresponding author.

## Ethics statement

The studies involving human participants were reviewed and approved by Human Ethics Committee of the Faculty of Arts and Science, Kyushu University. The patients/participants provided their written informed consent to participate in this study.

## Author contributions

JX, YJ, and JL contributed to the design of the study. JX and YJ programmed the experiments, conducted the data collection for the study, and analyzed the behavior. JX prepared all figures. JX and JL wrote the manuscript. All authors contributed to the article and approved the submitted version.

## Funding

This research was supported by project grant JP16H03751 (PI: JL) from the Japan Society for the Promotion of Science, and by a Ph.D. scholarship awarded to YJ by the Kyushu University SPRING program.

## Conflict of interest

The authors declare that the research was conducted in the absence of any commercial or financial relationships that could be construed as a potential conflict of interest.

## Publisher’s note

All claims expressed in this article are solely those of the authors and do not necessarily represent those of their affiliated organizations, or those of the publisher, the editors and the reviewers. Any product that may be evaluated in this article, or claim that may be made by its manufacturer, is not guaranteed or endorsed by the publisher.
